# mRNA localization to Pbodies in yeast is biphasic with many mRNAs captured in a late Bfr1pdependent wave

**DOI:** 10.1242/jcs.139055

**Published:** 2014-03-15

**Authors:** Clare E. Simpson, Jennifer Lui, Christopher J. Kershaw, Paul F. G. Sims, Mark P. Ashe

**Affiliations:** 1 Department of Biochemistry, Downing Site, The University of Cambridge, Cambridge CB2 1QW, UK; 2 The Faculty of Life Sciences, The Michael Smith Building, The University of Manchester, Oxford Road, Manchester M13 9PT, UK; 3 Faculty of Life Sciences, Manchester Institute of Biotechnology MIB, The University of Manchester, 131 Princess Street, Manchester M1 7DN, UK

**Keywords:** Pbodies, Stress granules, Glucose regulation, mRNA localization, Yeast

## Abstract

The relocalization of translationally repressed mRNAs to mRNA processing bodies Pbodies is a key consequence of cellular stress across many systems. Pbodies harbor mRNA degradation components and are implicated in mRNA decay, but the relative timing and control of mRNA relocalization to Pbodies is poorly understood. We used the MS2GFP system to follow the movement of specific endogenous mRNAs in live *Saccharomyces cerevisiae* cells after nutritional stress. It appears that the relocalization of mRNA to Pbodies after stress is biphasic some mRNAs are present early, whereas others are recruited much later concomitant with recruitment of translation initiation factors, such as eIF4E. We also find that Bfr1p is a latephaselocalizing Pbody protein that is important for the delayed entry of certain mRNAS to Pbodies. Therefore, for the mRNAs tested, relocalization to Pbodies varies both in terms of the kinetics and factor requirements. This work highlights a potential new regulatory juncture in gene expression that would facilitate the overall rationalization of protein content required for adaptation to stress.

## INTRODUCTION

Stringent regulation of mRNA translation and degradation is fundamental in allowing eukaryotic cells to control their diverse protein content. These mechanisms become especially important following stress cells must decrease their energy consumption while accumulating proteins that are required for adaptation Pichon et al., 2012 Simpson and Ashe, 2012. mRNA processing bodies Pbodies are induced under such stress conditions and represent sites where components of the 5 to 3 mRNA decay pathway are concentrated Buchan et al., 2010 Kedersha et al., 2005. As this pathway serves as a major route for bulk mRNA degradation, Pbodies are considered sites where mRNA can be degraded, particularly after stress Balagopal et al., 2012 Balagopal and Parker, 2009 Buchan et al., 2010. Pbodies also have key functions during embryonic patterning Weil et al., 2012, viral infection Beckham and Parker, 2008, microRNAmediated decay Pillai et al., 2005, and nonsense or AUrichelementmediated decay Durand et al., 2007 FengerGrn et al., 2005.

In addition to Pbodies, several other classes of mRNAcontaining granule have been identified. These include stress granules, which are found in many cell types, and neuronal granules and Pgranules, which have been found in neurons and embryonic cells, respectively Thomas et al., 2011 Updike and Strome, 2010. These granules occur in a variety of conditions and they contain many overlapping components, such as mRNAs, mRNAbinding proteins and proteins associated with translation inhibition Kedersha and Anderson, 2009 Parker and Sheth, 2007. Previously, we and others have characterized granules that harbour mRNA and translation initiation factors that accumulate as a response to stress in *Saccharomyces cerevisiae* Buchan et al., 2008 Hoyle et al., 2007. We studied the relocalization of the eukaryotic translation initiation factors, eIF4E, eIF4G and Pab1p both to Pbodies and to stressinduced granules EGPbodies, which contain these select mRNAassociated translation initiation factors but, crucially, lack the components of the mRNA decay machinery that are associated with Pbodies Hoyle et al., 2007. Other stress granules have been identified that form in response to very severe stress in yeast, and these granules are much more akin to mammalian stress granules, at least in terms of composition Grousl et al., 2009 Grousl et al., 2013 Kato et al., 2011. Therefore, the relationship between the various components of Pbodies and other RNA granules is complex and is thought to be dynamic, meaning proteins and mRNAs are able to move between granule subsets Buchan et al., 2010 Kedersha et al., 2005. For instance, studies have shown that mRNAs have the ability to reenter the translational pool following relief from stress Brengues et al., 2005 and that this ability might be specific to certain mRNAs, occurring over a finite period Arribere et al., 2011.

mRNAbinding proteins are well established to play crucial roles in determining mRNA fate and are associated with RNA granule formation. For instance, Edc3p, Pat1p and the Lsm17p binding complex function in Pbody regulation, whereas the RNAbinding protein TIA1 Pub1p in yeast is necessary for stress granule formation Decker et al., 2007 Duttagupta et al., 2005 Kedersha et al., 1999 Reijns et al., 2008. Furthermore, the tristetrapolin protein in mammalian cells is thought to regulate association of the AUrich cytokine mRNAs with Pbodies Franks and LykkeAndersen, 2007. The association of a variety of RNAbinding proteins with Pbodies not only suggests that many mRNAs are localized here, but also highlights the possibility that the localization of mRNAs might be differentially regulated. Although the targeting of mRNA to Pbodies has been studied for specific mRNAs Arribere et al., 2011 Hoyle et al., 2007 Lavut and Raveh, 2012 Sheth and Parker, 2006, key questions regarding the timing and specificity of mRNA recruitment remain unanswered.

In this study, we have used the mTAG system to investigate mRNA relocalization after stress in live yeast cells. Characterization of numerous mRNAs in this manner revealed that there are at least two distinct phases of mRNA localization to Pbodies. Firstly, there are those mRNAs that are present in RNA granules early after glucose starvation. Later, there is a second phase of mRNA recruitment to Pbodies after more extended periods of stress, which is reliant upon the earlier formation of Pbodies. A screen for Xrn1pDcp2p interacting proteins identified a Pbody protein, Bfr1p, that, similar to the late mRNAs, localizes to Pbodies in a delayed manner. Furthermore, deletion of the *BFR1* gene prevented the latephase entry of specific mRNAs to Pbodies suggesting that this mRNAbinding protein plays an important role in the regulation of mRNA fate following glucose depletion.

## RESULTS

### There are two mRNA localization profiles relative to Pbodies following glucose starvation

In order to inspect and follow the localization of specific mRNAs in live *S. cerevisiae* cells, we used the mTAG system Haim et al., 2007. A detailed explanation of this technique has been provided elsewhere HaimVilmovsky and Gerst, 2009 however, in short, the genomic copy of an mRNA sequence is tagged within its 3UTR with MS2 stem loops. This allows the visualization of the mRNA via coexpression of the MS2 coat protein fused to three green fluorescent proteins CPGFP_3_. Such systems have been used extensively to examine the localization of mRNAs in a wide range of biological systems Haim et al., 2007 Hamada et al., 2003 Sheth and Parker, 2006. Key advantages of this yeast system are that the control elements associated with mRNA transcription and processing promoters, UTRs, polyA sites and terminators remain intact, as the MS2binding sites are inserted directly and precisely into the 3UTR of the endogenous gene at its chromosomal locus Haim et al., 2007. A potential limitation of the approach is that the insertion of the MS2 stem loops could alter aspects of the behavior of an mRNA however, a number of mRNAs have been functionally evaluated after MS2 insertion and found to be unaffected Haim et al., 2007. Consequently, using this system, the localization of an mRNA can be evaluated in live cells, allowing responses to changing external cues to be assessed.

At the outset, we tagged numerous mRNA sequences with MS2 stem loops these mRNAs were selected because they are highly abundant and the protein products are associated with a variety of functions supplementary material Table S1. It might be predicted that the addition of the MS2 stem loops would increase the stability of the mRNA however, most of the resulting strains exhibited little difference in the expression level of the MS2tagged mRNAs relative to the level of untagged mRNA in wildtype strains as judged by quantitative reverse transcription qRTPCR. Levels of the *PGK1* and *RPS16A* mRNAs actually decreased with the added MS2 sequences supplementary material Fig. S1. It is currently unclear how this decrease in mRNA level occurs. Nevertheless, levels of the MS2tagged mRNAs being tested are either wild type or lower than this. The MS2tagged mRNA strains also contained the markers Dcp2pcyan fluorescent protein CFP and Cdc33pred fluorescent protein RFP. Dcp2p is the catalytic subunit of the decapping enzyme and serves as a marker for Pbodies, whereas Cdc33p is the eIF4E translation initiation factor that binds the mRNA cap and enters both Pbodies and EGPbodies stress granules. In these tagged strains, therefore, we define EGPbodies as harboring eIF4E but not the Pbody marker Dcp2p.

**Fig. 1.**
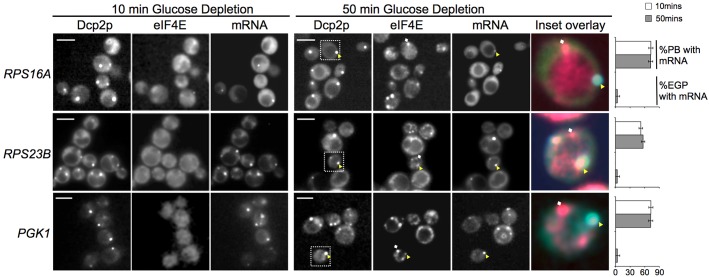
**Earlyphase mRNAs are present in Pbodies early after glucose depletion.** Fluorescence microscopy images of yeast cells at two different time points after glucose depletion. The *RPS16A*, *RPS23B* and *PGK1* mRNAs are followed using the mTAG system MS2GFP, mRNA decay components are followed using CFPtagged Dcp2p and components of the closed loop complex are followed using RFPtagged eIF4E across the same cells. The colored inset overlay images after 50minutes of glucose depletion depict examples where the mRNAs colocalize with Pbodies yellow triangles but not with EGPbodies white diamonds. Graphs to the right represent the percentage of Pbodies or EGPbodies that harbor each mRNA after 10minutes white bars and 50minutes gray bars of glucose depletion. Scale bars 5 m.

From the outset, it was evident that mRNAs fell into two categories of mRNA localization. The first class of mRNA observed using the mTAG system is characterized by mRNAs that colocalized with the Pbody marker Dcp2CFP early after Pbodies have formed, that is after 10minutes of glucose starvation Fig.1. These mRNAs also exhibited no real increase in the level of Pbody association from 10 to 50minutes post glucose starvation. Furthermore, there was little evidence that these mRNAs accumulated in granules that harbor eIF4E but lack Dcp2p, i.e. EGPbodiesstress granules. Examination of the localization of these mRNAs under nonstress conditions revealed that here too they were present in mRNA granules supplementary material Fig. S2. Under these nonstress conditions, neither the mRNA decay components nor the translation initiation factors exhibit any granular localization Hoyle et al., 2007 and data not shown. A detailed characterization and functional analysis of the mRNA granules that are present in exponentially growing cells will be published elsewhere. In this current study, we have focused on the localization of mRNAs to Pbodies and stress granules, and this class of mRNA enters Pbodies early after their formation. Two of these mRNAs, *RPS16A* and *RPS23B*, encode ribosomal proteins. A rapid targeting of mRNAs encoding ribosomal proteins to Pbodies, most likely for degradation, agrees with previous research showing that such mRNAs rapidly diminish in polysomes after glucose starvation Arribere et al., 2011. Another mRNA in this class is *PGK1*, which encodes the glycolytic enzyme phosphoglycerate kinase. Previously, using a plasmidU1Abased mRNAlocalization strategy, we have shown that the *PGK1* mRNA partially colocalized with eIF4E in granules Hoyle et al., 2007. Here, using the mTAG system in strains where both eIF4E and Dcp2p can be simultaneously visualized, we determined that the *PGK1*mRNAcontaining granules are Pbodies that also contained eIF4E Fig.1. Overall, this first class of mRNA is present early in Pbodies and does not localize to EGPbodiesstress granules.

**Fig. 2.**
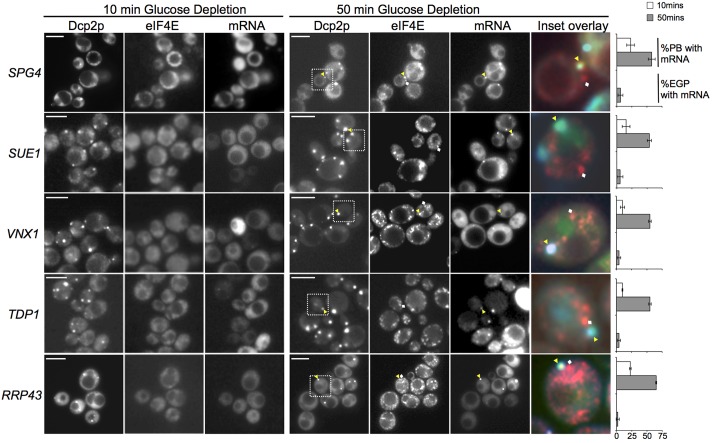
**Latephase mRNAs enter Pbodies after an extended period of glucose starvation.** As Fig.1, following *SPG4*, *SUE1*, *VNX1*, *TDP1* and *RRP43* mRNA relative to mRNA decay and closed loop complex components. The colored inset overlay images after 50minutes of glucose depletion depict examples where the mRNAs colocalize with Pbodies yellow triangles but not with EGPbodies white diamonds. Graphs to the right show that the percentage of Pbodies harboring each mRNA increases from 10minutes white bars to 50minutes gray bars of glucose depletion, whereas minimal colocalization with EGPbodies was observed. Scale bars 5 m.

We also identified a second class of mRNA that displayed different localization kinetics. These mRNAs were unlocalized both under nonstress conditions supplementary material Fig. S2 and after 10minutes of glucose depletion a time when Pbodies had already formed, as judged by the localization of Dcp2pCFP Fig.2. Therefore, at this early stage the majority of Pbodies lacked the tagged mRNA. However, after 50minutes of glucose starvation, localization of these mRNAs to Pbodies occurred Fig.2. As for the early class of mRNAs, there is little evidence that these mRNAs accumulated in granules that harbor eIF4E but lack Dcp2p, i.e. EGPbodiesstress granules. Thus, it appears that this second class of mRNA localizes to Pbodies over an extended time after stress, and does not localize to EGPbodies stress granules.

A protracted entry of mRNA into Pbodies is consistent with a current model for translational repression following glucose starvation Castelli et al., 2011. In this model, a loss of the eIF4A RNA helicase from the mRNA would inhibit translation initiation. However, over a short period after the stress, the eIF4A loss would cause the 48S preinitiation complex to accumulate. Over a more prolonged period, this stalled 48S complex would break up, allowing the slow release of mRNA associated with components of the closed loop complex. This could explain why eIF4E only localized to Pbodies at 50minutes, but not 10minutes, post glucose stress Fig.2 Hoyle et al., 2007. A more detailed quantification of the association of the MS2tagged mRNAs and eIF4E with Pbodies suggested that for all eight MS2tagged mRNAs evaluated in this study, only a small percentage of the tagged mRNA was present in Pbodies that also contain eIF4E supplementary material Fig. S3. Although it is still possible that mRNAs enter Pbodies as part of the closed loop complex, this quantification also highlighted an alternative possibility that the closed loop complex breaks down prior to, or during, the movement of these mRNAs to Pbodies.

**Fig. 3.**
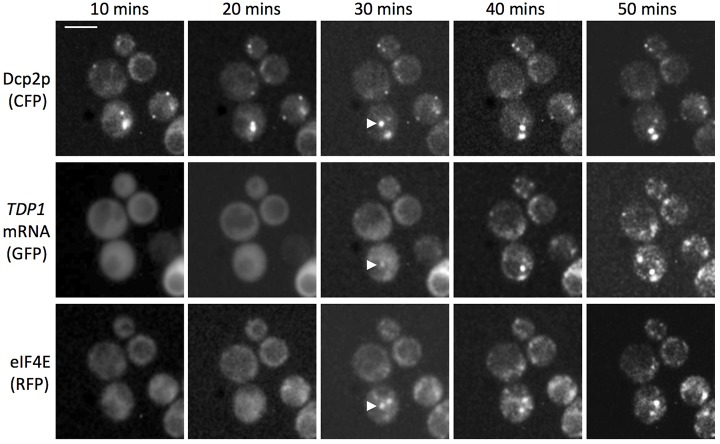
**Time course of localization of a latephase mRNA to Pbodies relative to eIF4E.** Fluorescence microscopy images of yeast cells over a time course after glucose depletion. The *TDP1* mRNA is followed using the mTAG system via MS2GFP middle row, mRNA decay components are followed using CFPtagged Dcp2p top row and components of the closed loop complex are followed using RFPtagged eIF4E bottom row. A white triangle highlights the time point where eIF4E and the mRNA are first observed colocalizing with the Pbody marker. Scale bar 5 m.

In order to compare the timing of eIF4E and latephase mRNA entry to Pbodies, a more comprehensive time course was performed using the *TDP1* mRNA. Here, the earliest time point at which localization to Pbodies is observable, for either eIF4E or the *TDP1* mRNA, was 30minutes post glucose starvation Fig.3. This apparent coincidence in the timing of movement for closed loop complex translation initiation factors and a latephase mRNA supports a model where at least some of the mRNA moves to Pbodies while still associated with the closed loop complex, following a protracted association with the translational machinery after glucose starvation. An obvious question from this comparison of late mRNA and eIF4E entry to Pbodies is why does eIF4E not move into Pbodies with the early mRNAs. A possible explanation is that the translationally repressed messenger ribonucleoprotein particle mRNP complex for the early mRNAs differs from that of the late mRNAs, and that, as a result, the mechanism of transfer to Pbodies differs.

### Localization of latephase mRNAs requires Pbodies

**Fig. 4.**
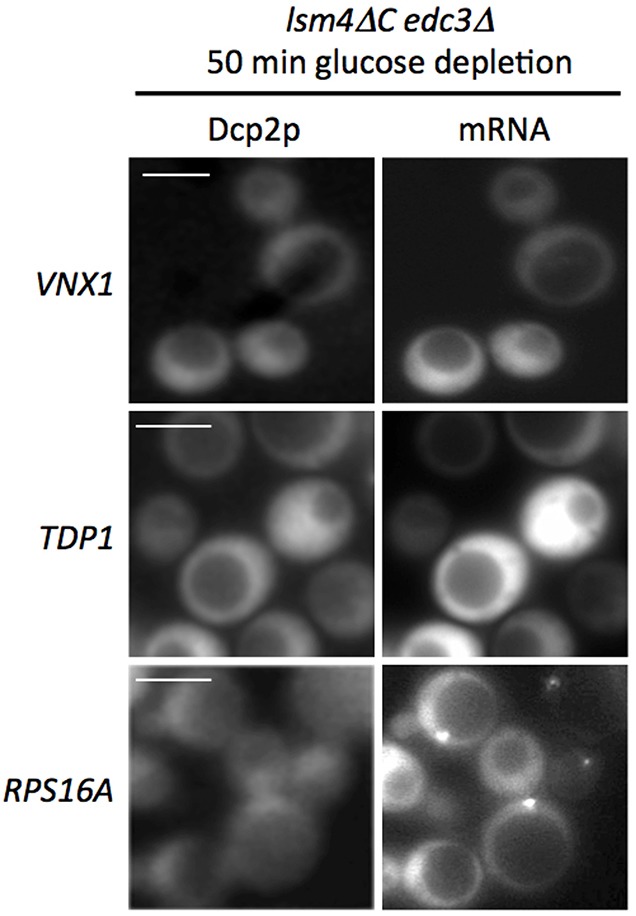
**Latephase mRNA localization relies upon Pbody formation.** Fluorescence images of yeast cells after 50minutes of glucose depletion. *lsm4C edc3* mutant strains were generated that carry the MS2tagged mRNAs labeled on the left. This mutant is deficient in Pbody formation, as shown by the lack of localization for Dcp2pCFP. The *RPS16A* mRNA provides an example where earlyphase mRNAs still aggregate, whereas localization is not observed for two latephase mRNAs, *VNX1* and *TDP1*. Scale bars 5 m.

In order to explore the mechanistic requirements for these two phases in mRNA recruitment to Pbodies, we made use of the *lsm4C edc3* mutant, which fails to form Pbodies. Lsm4p and Edc3p proteins contain specific domains that are essential for Pbody formation, presumably as a consequence of their potential for aggregation Decker et al., 2007. Therefore, strains bearing the various MS2tagged mRNAs were individually backcrossed to *lsm4C edc3* mutant strains. As expected, for all of the resulting *lsm4C edc3* mutant strains, Pbodies did not form after either 10 or 50minutes of glucose depletion Fig.4. Latephase mRNAs, such as *TDP1* and *VNX1*, were not localized at either early or late time points post glucose starvation in the mutant background. This result suggests that localization of these latephase mRNAs is dependent on the formation of Pbodies Fig.4. In contrast, *RPS16A* mRNA was observed in granules in the complete absence of Pbody formation. This observation most likely relates to the localization of these mRNAs to granules in unstressed cells, where Pbodies are not observed. These results do suggest that the distinction between the early and latephase mRNAs lies not only in the timing of the localization but also in the precise molecular mechanisms of the mRNA localization for each class of mRNA.

### Bfr1p is a late entry Pbody protein

In order to screen for factors copurifying with the mRNA decay factors Xrn1p and Dcp2p, we used tandem affinity purification TAP chromatography to pull down Dcp2pTAP and Xrn1pTAP from appropriate TAPtagged strains. Many interacting proteins were identified that have connections with RNA, or are associated with Pbodies these will be presented in greater detail elsewhere. A particularly prominent protein that was identified by mass spectrometry in these pull downs was Bfr1p Table1. Bfr1p is an mRNAbinding protein that was initially identified as a highcopy suppressor of the lactone antibiotic brefeldinA Jackson and Kps, 1994. More recent studies have shown that Bfr1p interacts with an mRNAbinding complex containing RNAbinding proteins, such as Scp160p Lang et al., 2001 Scheuner et al., 2001 Sezen et al., 2009. Both Scp160p and Bfr1p colocalize with the yeast endoplasmic reticulum ER in a characteristic pattern around the nucleus cortical ER and just under the cell membrane peripheral ER Mitchell et al., 2013 Sezen et al., 2009. The Bfr1pcontaining complex has also been shown to localize to polysomes in exponentially growing cells, suggesting that it is actively involved in regulating translation Sezen et al., 2009. On this basis, the potential interaction between Bfr1p and mRNA decay factors present in Pbodies was further investigated.

**Table 1.**
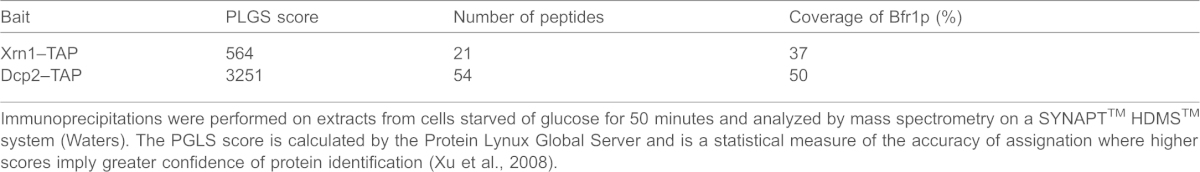
Summary of Bfr1p identification via mass spectrometry in affinity purifications of Dcp2p and Xrn1p

Immunoprecipitations were performed on extracts from cells starved of glucose for 50minutes and analyzed by mass spectrometry on a SYNAPT^TM^ HDMS^TM^ system Waters. The PGLS score is calculated by the Protein Lynux Global Server and is a statistical measure of the accuracy of assignation where higher scores imply greater confidence of protein identification Xu et al., 2008.

**Fig. 5.**
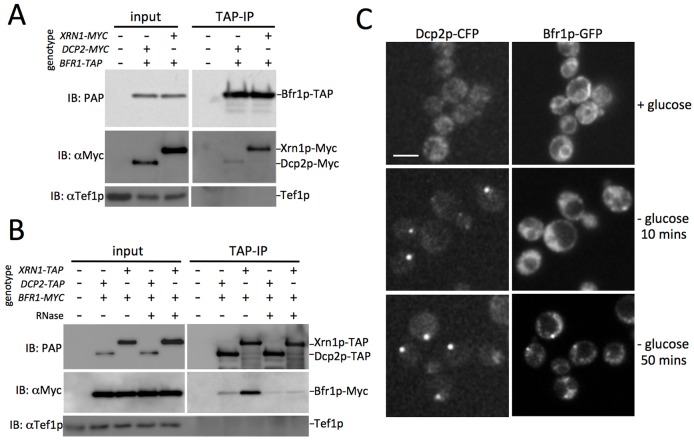
**Bfr1p interacts with Dcp2p and Xrn1p via RNA and enters Pbodies.** A Western blots IB from TAP TAPIP on strains bearing TAPtagged Bfr1p, as well as Myctagged Xrn1p or Dcp2p. B The reciprocal affinity purification of either Xrn1pTAP or Dcp2pTAP in strains containing Bfr1pMyc. In both A and B, TAPtagged proteins were detected with a protein A peroxidase conjugate PAP, Myctagged proteins were detected with an antibody against Myc, the presence of Tef1p was detected using an antibody against Tef1p. In both sets of experiments, an untagged wildtype strain was used as a negative control on the same gel. C Localization of Bfr1p in exponentially growing cells glucose or after 10 or 50minutes of glucose starvation glucose. Scale bar 5 m.

TAPaffinity purifications on extracts prepared from strains harboring Bfr1pTAP revealed that Myctagged Xrn1p, and a small amount of Myctagged Dcp2p, could be immunoprecipitated with Bfr1p Fig.5A. The eEF1A translation elongation factor Tef1p serves as a specificity control, as this protein is one of the most abundant in the cell and is often a contaminant of immunoprecipitations Krogan et al., 2006. Therefore, the absence of this factor demonstrates the specificity of the interactions between Bfr1p and the mRNA decay factors. The reciprocal purification of either Xrn1pTAP or Dcp2pTAP also resulted in copurification of Myctagged Bfr1p Fig.5B. Given that both Bfr1p and Xrn1pDcp2p have previously been described as being associated with mRNA, or have been implicated in mRNA degradation, the RNA dependence of the interaction was assessed by treatment with RNase I. This treatment results in a reduction in the level of coimmunoprecipitation suggesting that most of the interaction occurs via RNA Fig.5B.

As Bfr1p has been described as being associated with mRNAs in polysomes, most likely at the ER Mitchell et al., 2013 Sezen et al., 2009 and, here, we show that it interacts via mRNA with the mRNA decay machinery, we considered the possibility that this protein could act as a mediator involved in the transition from mRNA translation to mRNA degradation. Such a mediator might be expected to accumulate at the site of mRNA degradation. In order to directly assess this, Bfr1p was GFPtagged using a genomic tagging strategy Janke et al., 2004. In exponentially growing cells, GFPtagged Bfr1p was observed both at the periplasm around the nucleus and at the cell periphery Fig.5C, consistent with previous studies that demonstrate an ER localization Huh et al., 2003 Mitchell et al., 2013. A more diffuse fluorescence was also observed throughout the cellular cytoplasm Fig.5C. After 10minutes of glucose starvation, the pattern of Bfr1p localization became more diffuse, whereas after 50minutes of glucose starvation, Bfr1p was observed to start to accumulate in Pbodies Fig.5C. Intriguingly, this delayed relocalization corresponded with that observed for the latephase mRNAs and the closed loop translation initiation factors, highlighting a possible role for Bfr1p in the transition of the latephase mRNAs from the translated pool to Pbodies.

### Bfr1p is necessary for latephase targeting of mRNAs to Pbodies

**Fig. 6.**
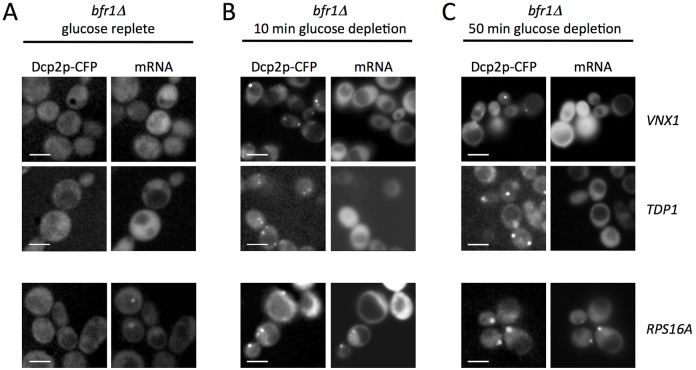
**Latephase mRNA localization to Pbodies requires Bfr1p.** Images of *bfr1* mutant strains containing Dcp2pCFP and MS2tagged *RPS16A*, *VNX1* or *TDP1*. The mRNA and Dcp2p localization is shown after A no starvation, B 10minutes of glucose starvation, C 50 minutes of glucose starvation. Scale bars 5 m.

In order to directly evaluate the role of Bfr1p in the transition of mRNAs to Pbodies, the localization of specific mRNAs was assessed in *bfr1* mutant strains. In all cases, following glucose starvation, Pbody formation was still observed at early time points Fig.6A,B. The *RPS16A* mRNA, which localizes to Pbodies almost immediately after their formation, was still observed in Pbodies Fig.6B,C. In contrast, two different latephase mRNAs, *VNX1* and *TDP1*, had not entered Pbodies after 50minutes of glucose starvation Fig.6B,C, or even after more protracted starvation periods data not shown. Therefore, the Bfr1p mRNAbinding protein is essential for the targeting of these latephase mRNAs to Pbodies. This difference between late and early mRNAs, in terms of their factor requirements, further exemplifies the mechanistic distinction between these two classes of mRNA.

## DISCUSSION

Cells change and adapt their proteome to cope with alterations that occur in their surroundings. This is true for both the simple unicellular organisms, like yeast, and cells from more complex multicellular organisms Simpson and Ashe, 2012 Spriggs et al., 2010 Toone and Jones, 1998 Welch, 1987. For instance, to survive stresses, such as nutrient depletion, cells must reduce their energy consumption, while rationalizing their protein content to adapt, in the shortest possible time, to the changing conditions. In *S. cerevisiae*, as a response to glucose depletion, energy consumption is minimized by a rapid downregulation of a variety of energyconsuming processes, including protein synthesis Ashe et al., 2000, actin polymerization Uesono et al., 2004, tRNA nucleocytoplasmic export Whitney et al., 2007 and </emph>endosomal trafficking Aoh et al., 2011. In particular, the rapid inhibition of translation initiation and subsequent appearance of Pbodies, combined with transcriptional reprogramming has been viewed as a means by which cells can rapidly alter their gene expression profile Arribere et al., 2011 Lui et al., 2010. In this study, we provide further evidence for the rationalization of mRNAs after stress by showing that there are at least two phases in the mobilization of mRNA to Pbodies. We show that these two phases have differing requirements, both in terms of Pbody formation and the mRNAbinding protein Bfr1p, which highlights the intriguing possibility that the phases could be independently controlled.

**Fig. 7.**
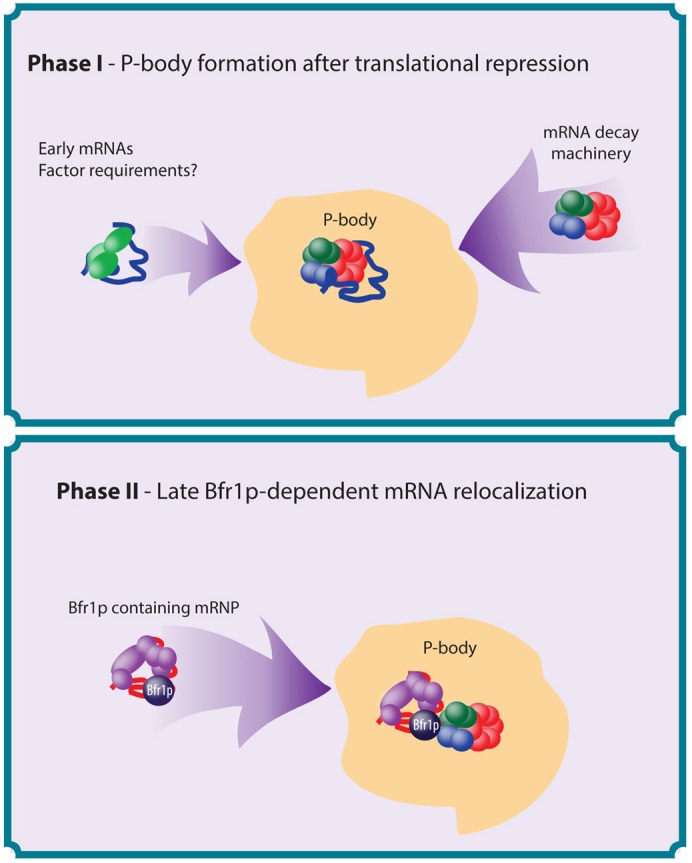
**A model depicting the two phases of mRNA relocalization to Pbodies.** Phase I. Following cellular stress, such as glucose starvation, earlyphase mRNAs relocalize to Pbodies with the mRNA decay machinery. Here, the mRNAs are either degraded or held in a translationally repressed state. Phase II. More prolonged glucose starvation leads to a release of latephase mRNAs that have been associated with the translation initiation machinery in a repressed state. These mRNAs are relocalized to Pbodies in a Bfr1pdependent manner.

Following glucose starvation, translation initiation is rapidly inhibited and, as a consequence, Pbodies form almost immediately. Typically, the first class of mRNA that we have identified colocalized with Pbody components instantly after Pbody formation. In contrast, a second class of mRNA localized to Pbodies gradually over an extended period of glucose starvation. More detailed kinetics reveal that the localization of mRNA coincides with the timing of eIF4E, eIF4G and Pab1p relocalization, which takes longer than 30minutes Hoyle et al., 2007. We have previously shown that glucose starvation leads to a rapid loss of the RNA helicase eIF4A from the translation machinery and the accumulation of a 48S intermediate complex lacking eIF4A Castelli et al., 2011. This complex persists for over 30minutes after glucose starvation, gradually decaying such that the mRNA, and the translation factors closely associated with it, might only be available to localize to Pbodies at later time points. Combined with the data presented here, this leads to a model where the latephase mRNAs localize to Pbodies gradually because of their prolonged association with the translation machinery. This could represent a mechanism by which the latephase mRNAs remain translationally primed, such that translation is inhibited without the mRNA being rapidly targeted for decay. This would allow translational resumption should conditions become more favorable. Longer periods of stress would allow the dissociation of the mRNA closed loop complex, either in the Pbody or during relocalization, such that the mRNA could be degraded Fig.7.

A precedent exists in terms of altered localization to Pbodies over time for the catalytic subunits of the cAMPdependent protein kinase PKA, Tpk2p and Tpk3p. Tpk2p localizes to Pbodies directly after glucose starvation with levels increasing over time, whereas Tpk3p localization only occurs at later time points, concurrent with the second phase of mRNA localization Tudisca et al., 2012. Intriguingly, PKA activity has also been linked to Pbody formation, in that the active PKA in glucosereplete cells supresses Pbody formation via phosphorylation of Pat1p and, after glucose starvation, dephosphorylation of Pat1p coincides with Pbody formation Ramachandran et al., 2011. Therefore, one possibility is that the regulated localization of the PKA catalytic subunits could play a role in the control of mRNA mobilization to Pbodies

The distinct nature of the two phases of mRNA localization to Pbodies is further reflected by two additional findings. Firstly, mutations that prevent Pbody formation do not impede the localization of the earlyphase mRNAs, whereas these mutations do prohibit the localization of the latephase mRNAs. Secondly, deletion of the *BFR1* gene prevents latephase mRNA localization without affecting the earlyphase mRNAs, but in this case Pbodies form normally. Bfr1p has previously been suggested to play a role in the inhibition of mRNA translation via Scp160p and its interaction with the eIF4Ebinding protein 4EBP Eap1p Lang et al., 2001 Sezen et al., 2009. Taken together with the data in our study, this provides evidence that Bfr1p might function as part of an intermediary mRNP complex that directs latephase mRNAs from the translation machinery to the mRNA decay system Fig.6. Studies aimed at defining the mRNAs bound by a range of RNAbinding proteins suggest that Bfr1p and Scp160p interact with in excess of 1000 mRNAs Hogan et al., 2008. Hence, it is possible that although Bfr1p is required for latephase entry to Pbodies, it does not provide the specificity with which mRNAs are selected for this fate. However, the exact nature and composition of the late and early mRNPs that enter Pbodies is unknown and will provide a focus for further studies.

Glucose starvation in yeast has also been shown to cause the appearance of granules that harbor translation initiation factors and RNAbinding proteins but lack the mRNA decay machinery Buchan et al., 2008 Hoyle et al., 2007. These have been termed both EGPbodies or stress granules. Other severe stress conditions have identified granules in yeast that are more similar to mammalian stress granules, as they harbor eIF3 and the 40S ribosomal subunit Grousl et al., 2009 Grousl et al., 2013 Kato et al., 2011. Of the mRNAs tested in this study, neither early nor latephase mRNAs appear to localize to EGPbodies following glucose starvation, instead these mRNAs are almost exclusively localized to Pbodies. Hence, even where the mRNA granule colocalizes with a translation initiation factor, such as eIF4E, this granule will also contain components of the mRNA decay machinery, such as Dcp2p. Therefore, we rarely observe the mRNA in granules that do not contain the Pbody markers after glucose starvation. There are several possible explanations for these observations mRNAs might not enter EGPbodies at all, they might enter for a very transient period, or a very specific subset of mRNAs might be present there. EGPbodies contain those translation initiation factors that are known to interact with mRNA, as well as at least three wellcharacterized RNAbinding proteins Buchan et al., 2011. Therefore it seems likely that mRNA is also a component of these granules and there is no reason to suspect that the mRNA would rapidly exit, or become degraded in a stress granule, as the mRNA decay machinery is absent. Therefore, we favor an explanation where only specific mRNAs are localized, although an example of such an mRNA has yet to be identified.

The formation of Pbodies provides an opportunity for cells to target their mRNA content for mRNA decay andor storage. The fact that individual mRNAs are recruited to these granules in distinct phases, which have distinct cofactor requirements, suggests that this process is more complicated than previously anticipated. Ultimately, this high degree of finetuning, in terms of the regulation of mRNA movement to granules, suggests that cells follow a precisely determined program of reorganization after stress. It is possible that each facet of this reorganization process would hold an evolutionary advantage in terms of cell survival.

## MATERIALS AND METHODS

### Strains and plasmids

*S. cerevisiae *strains used in this study are listed in supplementary material Table S2. Proteins were tagged at the Cterminus and verified by PCR analysis Campbell and Ashe, 2007. MS2binding sites were inserted into 3UTRs and verified using PCR and RTPCR Fig.1 Haim et al., 2007. Knockout strains were generated using a KanMX2 insertion cassette Wach et al., 1994 and verified using PCR on genomic DNA samples. The *edc3 lsm4C* mutant kindly provided by J. Hasek Grousl et al., 2009 was backcrossed four times to W3031A, then backcrossed to the MS2Ltagged strains to generate *edc3 lsm4C DCP2CFP* MS2LmRNA strains.

### Growth conditions

Cells were grown at 30C to OD_600_ 0.4 in synthetic complete medium with 2 glucose SCD Guthrie and Fink, 1991. Cells were incubated for 1hour in SCD medium lacking methionine to induce expression of pCPGFP_3_. For stress conditions, cells were incubated in medium lacking glucose SC for 10minutes or 50minutes.

### Microscopy and quantification

Epifluorescent images used for quantification were acquired on an Eclipse E600 microscope using a 1001.40 numerical aperture oil plan Apo objective. Images were collected using Axiovision 4.5 software Carl Zeiss MicroImaging, Inc. and camera. Representative cells are shown from experiments repeated at least three times. Granules were counted using 100 cells for each mRNA in triplicate. All other images were taken using the delta vision RT Applied Precision with a 1001.40 numerical aperture differential inference contrast oil plan Apo objective Olympus and camera CoolSNAP HQ Roper Scientific using Softworx 1.1 software Applied Precision and 11 binning.

### Quantitative realtime reverse transcriptase PCR

RNA analysis by quantitative reverse transcription PCR qRTPCR was carried out using the iScript^TM^ onestep RTPCR kit with SYBR green BioRad on a CFX connect^TM^ realtime PCR detection system BioRad. The primers used are listed in supplementary material Table S3 and signals were quantified relative to actin mRNA using the 2^Ct^ method Livak and Schmittgen, 2001.

### Protein analysis

TAP tag purification experiments were completed using metal tosylactivated dynabeads Invitrogen bound to 10mgml IgG. 10mg of total protein prepared from exponentially growing yeast cells was incubated with the beads for 20minutes. Where samples were RNase treated, 100l RNase I Ambion was added during the incubation with beads. Beads were washed five times in buffer 20mM TrisHCl pH8, 140mM NaCl, 1mM MgCl_2_, 0.5 NP40, 0.5mM DTT, 1mM phenylmethylsulfonyl fluoride. Bound protein was analyzed by western blotting. TAPtagged proteins were detected using horseradish peroxidase HRPconjugated protein A PAP Abcam, or 9Myc proteins were detected using a Myc antibody Millipore and Tef1p was detected using an endogenous antibody a gift from Chris Grant.

### Proteomic analyses

Cells were grown at 30C to an OD_600_ of 0.8 and depleted of glucose for 50minutes, as for the microscopy and western blotting analysis. Bound proteins were eluted from the IgG Dynabeads Invitrogen using sequential solutions of 0.5 M acetic acid then 500mM hydrogen peroxide. Whole eluates were dried down and resuspended in a solution of 80 acetonitrile, 20 50mM TrisHCl pH7.6, 10mM CaCl_2_ and 250ng trypsin. Samples were incubated for 1hour at 37C, then dried down and resuspended in 10 acetonitrile and 0.1 formic acid for mass spectrometry analysis. Mass spectrometry analysis was performed using the SYNAPT HDMS^TM^ Waters mass spectrometer followed by analysis using PGLS analysis software.

## Supplementary Material

Supplementary Material
