# Supplementary material for: mRNA localization to Pbodies in yeast is biphasic with many mRNAs captured in a late Bfr1pdependent wave
Source: J Cell Sci. 2014 Mar 15;127(6):1254–62. doi: 10.1242/jcs.139055 (PMC3953815; doi:10.1242/jcs.139055)
Supplement: Supplementary Material [file supp_127_6_1254__index.html]

mRNA localization to Pbodies in yeast is biphasic with many mRNAs captured in a late Bfr1pdependent wave — Supplementary Material 

# mRNA localization to Pbodies in yeast is biphasic with many mRNAs captured in a late Bfr1pdependent wave

## JCS139055 Supplementary Material

**Files in this Data Supplement:**

- **Supplementary Material**
