# Supplementary material for: mRNA localization to Pbodies in yeast is biphasic with many mRNAs captured in a late Bfr1pdependent wave
Source: J Cell Sci. 2014 Mar 15;127(6):1254–62. doi: 10.1242/jcs.139055 (PMC3953815; doi:10.1242/jcs.139055)
Supplement: Supplementary Material [file supp_127.6.1254_JCS139055.pdf]

Simpson et al., Supplementary Fig. S1

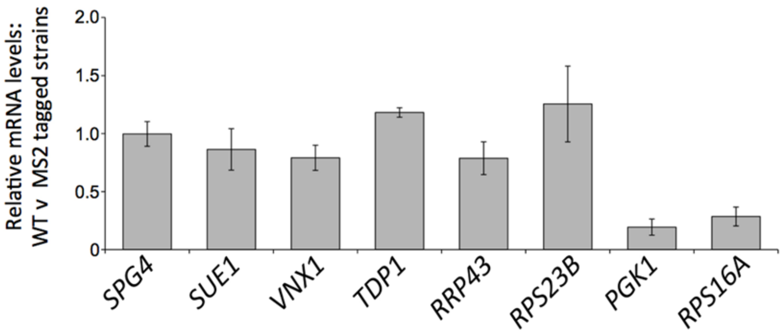

Expression levels of MS2 tagged versus untagged mRNAs. Results show the mean fold change in the labelled mRNA in the MS2-tagged strain relative to wild type. The error bars represent the standard error of the mean.

Simpson et al., Supplementary Fig. S2

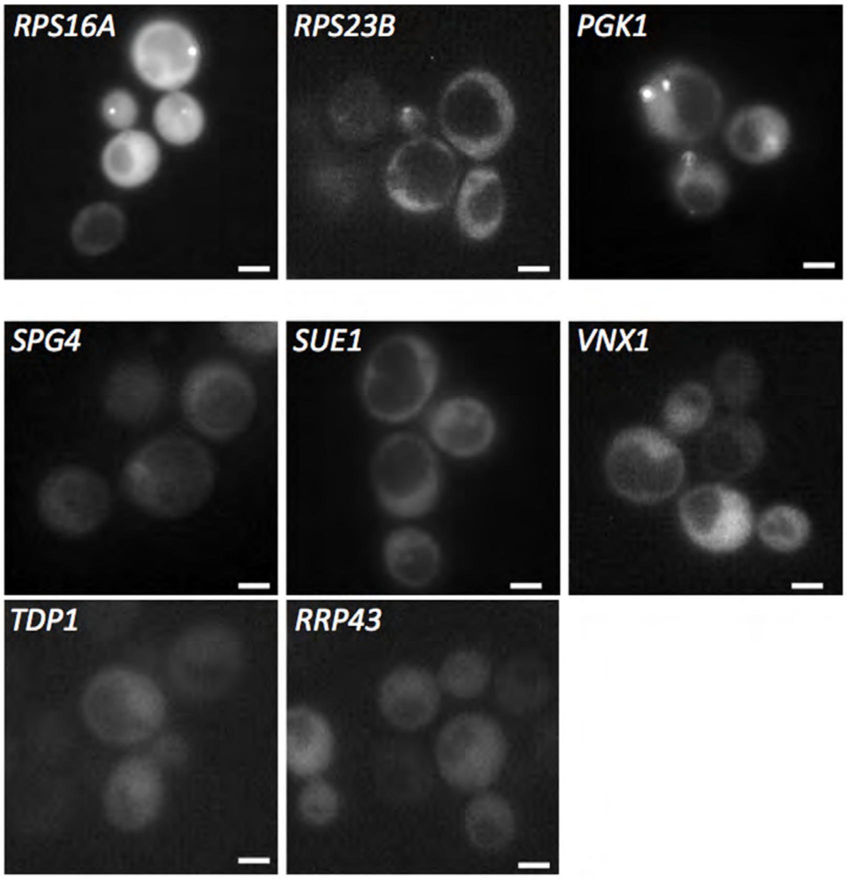

mRNA localisation in exponentially growing cells. mRNAs were visualised using the m-TAG system in unstressed cells grown at 30ºC to OD 600 0.4 in synthetic complete medium with 2% glucose (SCD) (Bar = 2μm).

Simpson et al., Supplementary Fig. S3

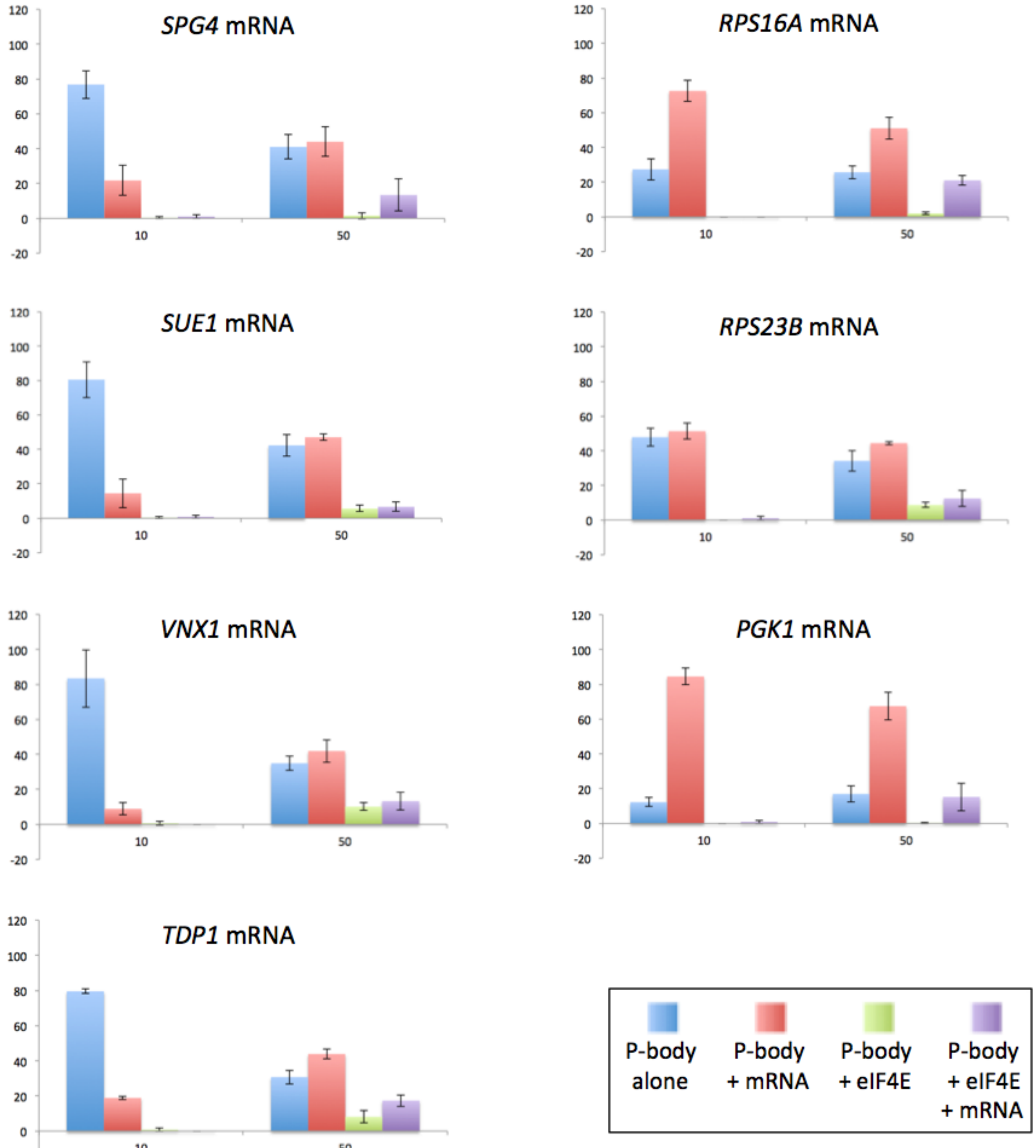

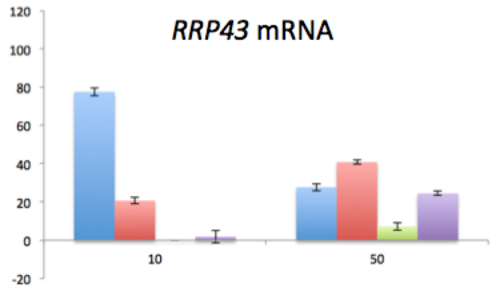

Detailed quantitation of and eIF4E localisation to P-bodies. Granules were counted using 100 cells for each strain across three biological replicates.

Table S1. List of selected genes and the functions of their protein products

Gene	Protein	Function
SPG4 (*yMR107w*)	Stationary phase protein	Protein required for survival at high temperature during stationary phase
SUE1 (*yPR151c*)	Mitochondrial protein	Protein required for degradation of unstable forms of cytochrome c
VNX1 (*yNL321w*)	Vacuolar monovalent cation/ H^+ antiporter	Promotes intracellular monovalent cation sequestration via exchange for hydrogen ions (especially Na^+across the vacuolar membrane)
TDP1 (*yBR223c*)	Tyrosyl-DNA phosphodiesterase	Tyrosyl-DNA phosphodiesterase I; hydrolyzes 3' and 5'-phosphotyrosyl bonds; involved in the repair of DNA lesions created by topoisomerases
RRP43 (*yCR035c*)	Exosome sub-unit	Exosome non-catalytic core component; roles in 3'-5' RNA processing and degradation
RPS23B (*yPR132w*)	40S Ribosomal protein 23B	Component of the small ribosomal subunit
RPS16A (*yMR143w*)	40S Ribosomal protein 16A	Component of the small ribosomal subunit
PGK1 (*yCR012w*)	3-phosphoglycerate kinase	Key enzyme in glycolysis and gluconeogenesis

Table S2. Yeast strains used in this study

Strain	Genotype	Source
yMK467	*MATα ADE2 his3-11, 15 leu2-3, 112 trp1-1 ura3-1*	Ashe Lab
yMK1725	yMK467 *lsm4ΔC::LEU2 edc3::URA3*	Ashe Lab
yMK2026	yMK467 *DCP2-TAP::TRP1 XRN1-MYC::HIS3*	This study
yMK2027	yMK467 *XRN1-TAP::TRP1 DCP2-TAP::HIS3*	This study
yMK2028	yMK467 *CDC33-RFP::NAT DCP2-CFP::TRP1 SUE1-MS2 p[CP-GFP$_3$ HIS3 CEN]*	This study
yMK2030	yMK467 *CDC33-RFP::NAT DCP2-CFP::TRP1 VNX1-MS2 p[CP-GFP$_3$ HIS3 CEN]*	This study
yMK2031	yMK467 *CDC33-RFP::NAT DCP2-CFP::TRP1 TDP1-MS2 p[CP-GFP$_3$ HIS3 CEN]*	This study
yMK2033	yMK467 *CDC33-RFP::NAT DCP2-CFP::TRP1 RPS23B-MS2 p[CP-GFP$_3$ HIS3 CEN]*	This study
yMK2034	yMK467 *CDC33-RFP::NAT DCP2-CFP::TRP1 PGK1-MS2 p[CP-GFP$_3$ HIS3 CEN]*	This study
yMK2035	yMK467 *CDC33-RFP::NAT DCP2-CFP::TRP1 RPS16A-MS2 p[CP-GFP$_3$ HIS3 CEN]*	This study
yMK2078	yMK467 *CDC33-RFP::NAT DCP2-CFP::TRP1 RRP43-MS2 p[CP-GFP$_3$ HIS3 CEN]*	This study
yMK2029	yMK467 *CDC33-RFP::NAT DCP2-CFP::TRP1 SPG4-MS2 p[CP-GFP$_3$ HIS3 CEN]*	This study
yMK2039	yMK467 *lsm4ΔC::LEU2 edc3::URA3 DCP2-CFP::TRP1 VNX1-MS2 p[CP-GFP$_3$ HIS3 CEN]*	This study
yMK2043	yMK467 *lsm4ΔC::LEU2 edc3::URA3 DCP2-CFP::TRP1 TDP1-MS2 p[CP-GFP$_3$ HIS3 CEN]*	This study
yMK2047	yMK467 *lsm4ΔC::LEU2 edc3::URA3 DCP2-CFP::TRP1 RPS16A-MS2 p[CP-GFP$_3$ HIS3 CEN]*	This study
yMK2079	yMK467 *XRN1-TAP::TRP1 BFR1-MYC::HIS3*	This study
yMK2080	yMK467 *DCP2-TAP::TRP1 BFR1-MYC::HIS3*	This study
yMK2044	yMK467 *XRN1-MYC::HIS3 BFR1-TAP::TRP1*	This study
yMK2045	yMK467 *DCP2-MYC::HIS3 BFR1-TAP::TRP1*	This study
yMK2046	yMK467 *CDC33-RFP::NAT DCP2-CFP::TRP1 TDP1-MS2 bfr1::G418 p[CP-GFP$_3$ HIS3 CEN]*	This study
yMK2040	yMK467 *CDC33-RFP::NAT DCP2-CFP::TRP1 RPS16A-MS2 bfr1::G418 p[CP-GFP$_3$ HIS3 CEN]*	This study
yMK2081	yMK467 *CDC33-RFP::NAT DCP2-CFP::TRP1 VNX1-MS2 bfr1::G418 p[CP-GFP$_3$ HIS3 CEN]*	This study
yMK2036	yMK467 *BFR1-GFP::G418 DCP2-CFP::TRP1 CDC33-RFP::NAT*	This study

Table S3. Oligonucleotides used in this study

Oligonucleotide	Sequence (5'-3')
ACT1 FW	AGAGATTTGACTGACTACTTGATG
ACT1 RV	GAAGATTGAGCAGCGGTTTG
PGK1 FW	TTGATTGACAACTTGTTGGA
PGK1 RV	CAGTGACAGTCTTGGTGTTG
RRP43 FW	AGTCTTGAGTCGCACTGGTCC
RRP43 RV	GGTGGCACTTCTTCCTCTTGTTC
RPS16A FW	ATGGTTCTCCAATCACTTTG
RPS16A RV	TAAACTTGGGAAACATGACC
RPS23B FW	TCTCCATTCGGTGGTTCTTC
RPS23B RV	ATCGTTTGGAACGAAAGCAG
SPG4 FW	GTTTTTGGGACGCATTCGCA
SPG4 RV	TGATGTGTCCTAGGTTGACGGT
SUE1 FW	ATGCGCATTTCTATGACTTT
SUE1 RV	TTTTTCTCATCGATCGTCTT
TDP1 FW	GGAACTAAGAGGAAGAGGTCGGATG
TDP1 RV	CGCAGTCGTCGCTATCATTGTTAC
VNX1 FW	CCCTGCAACGGAATCTTCGT
VNX1 RV	AAGTACCGTCTTTGAGCCGC
